# Collectanea Medica: Consisting of Anecdotes, Facts, Extracts, Illustrations, &c.

**Published:** 1823-09

**Authors:** 


### COLLECTANEA MEDICA:

CONSISTING OF

### ANECDOTES, FACTS, EXTRACTS, ILLUSTRATIONS, &c.

*Relating to the History or the Art of Medicine, and the Collateral Sciences.*

Floriferis, ut apes, in saltibus omnia libant,

Omnia nos, itidem, depascimur aurea dicta.

---

ART. I.—*Extracts from "Medical Jurisprudence."* By J. A. PARIS, M.D. &c. and J. S. M. FONBLANQUE, Esq.

### *Of Parturition, or Delivery.*

THE term of utero-gestation is limited by nature to nine calendar months, or forty weeks, at the expiration of which the process of labour usually commences. Ingenious theorists have endeavoured to discover the principle of the expulsatory action of the uterus, and to assign the reason of its taking place at a stated period; but, after all the subtle ingenuity which has been displayed upon this occasion, it is doubtful whether we are prepared with a better solution of the problem than that furnished by the physiologist in the time of Avicenna, who declared that labour came on at the appointed season, *by the command of God.* We shall therefore pass over the question without farther discussion, and proceed to the investigation of those practical parts of the subject which are highly interesting on account of their numerous and important relations to medical jurisprudence.

1. *Whether a woman can be delivered during a state of insensibility, and remain unconscious of the event?*

In certain comatose states of the brain, as those produced by depression of bone, the operation of narcotic substances, or the violence of fever, we must admit the possibility of such an occurrence. Hippocrates relates the case of a woman who was delivered during a state of insensibility in the last stage of fever, from which she never recovered, and therefore died unconscious of the event. In the "Causes Celèbres," the case of the Comtesse de Saint Geran is recorded, who, having been plunged into a profound sleep by a medicated draught prepared for that

---

purpose, brought forth a son without being in the least conscious of the act that gave it birth; and, when she awoke on the following day, bathed in her blood and exhausted in strength, and demanded her infant, the artful attendants denied the fact of her delivery. Women have moreover given birth to an offspring in *articulo mortis;* and many instances have occurred where the infant has escaped from the womb during the exertions of the mother to evacuate the contents of the bowels.

2. *How far the term of utero-gestation can be shortened, to be compatible with the life (viabilité) of the offspring?*

If this question could be decided by the number of recorded cases, we should be called upon to acknowledge the possibility of the fœtus surviving at extremely early periods. Capuron relates the case of Fortunio Liceti, who, it is said, was born at the end of four months and a half, and that he lived to complete his twenty-fourth year! In the case of Marechal de Richelieu, the parliament of Paris decreed that the infant at five months possessed that capability of living to the ordinary period of human existence *(viabilité,)* which the law of France required for establishing its title of inheritance. The Roman law, "*de suis et legitimis hæredibus,*" establishes, upon the authority of Hippocrates, that an infant may be born six months and two days after the term of conception; while a second law, sanctioned also by the same high authority, requires an interval of seven months between the conception and delivery. This discrepancy receives explanation from the fact that the ancients fell into many contradictions from indiscriminately using in their calculations lunar and solar months: thus, for instance, Hippocrates uses the former in his books "de Septimestri et Octomestri partu," while in those "de Alimento," "de Carnibus," "de Epidemicis," the latter uniformly constitute the basis of computation.

Physiologists of the present day consider that a fœtus born before the completion of the seventh month has a very slender chance of surviving, although instances have occurred where the life has been preserved after a birth still more premature. Hippocrates and other ancient physicians entertained a conceit, which has even prevailed in the more modern schools of physic, that an infant could live at seven, but rarely or never at eight, months. It is hardly necessary to observe, with Haller, that the capability of living in an infant increases in the ratio of its maturity, or in proportion as it advances towards the natural period of delivery: the child, therefore, that is born at the expiration of eight months has, of necessity, a greater aptitude for living than the one which is produced at seven; and nothing could have suggested or upheld a contrary opinion, but that overwhelming belief in the harmony and powers of certain numbers with which the philosophers of ancient days were infected, and of which the Pythagorean number *seven* was deemed the most perfect and efficient, as we have before had occasion to remark while treating of the subject of Ages.

3. *Whether to any, and to what probable extent, the natural term of utero-gestation can be protracted?*

Although the period of gestation is usually limited to nine calendar months, or forty weeks, yet the term does not appear to be so arbitrarily established, but that nature may occasionally transgress her usual law; and, as we have just stated that many circumstances may occur to anticipate delivery, so are we bound to admit that in some instances it may be retarded. In several tolerably-well attested cases, the birth appears to have been protracted several weeks beyond the time of delivery; and Dr. Hamilton remarks upon this occasion, that, if the character of the woman be unexceptionable, a favourable report should be given for the mother, though the child should not be produced till nearly ten calendar months after the absence or sudden death of her husband. The question is one of the greatest importance in its moral and legal relations, for it may involve the honour and happiness of families, the legitimacy of offspring, and the succession of property. We cannot, therefore, feel surprised that it should have occupied so great a portion of the attention of our most able physiologists, and have given origin to considerable controversy. Each side is supported by an equally respectable list of partisans, and we see that upon this occasion the two celebrated medico-jurisconsults of France are opposed to each other: Mahon having associated his name with those of Bohn, Hebenstreit, Astruc, Mauriceau, De La Motte, Ræderer, and Baudelocque, who reject the belief in retarded delivery as impossible, and contrary to the immutable law of nature; while the name of Foderé ranges with those who support the contrary opinion, as Teichmeyer, Heister, Albert, Vallentini, Bartholin, Haller, Antoine Petit, Lietaud, Vicq d'Azyr, and Capuron, who may boast of the support of Hippocrates, Aristotle, and Pliny. * * *

The civil code of France has placed a limit to our credulity respecting retarded births, and decrees three hundred days, or ten months, to be the most distant period at which the legitimacy of a birth shall be allowed. By the law of Scotland, a child born six months after the marriage of the mother, or ten months after the death of the father, is considered as legitimate.

4. *What is the value of those signs by which we seek to establish the fact of a recent delivery?*

There are circumstances which may induce a woman to conceal the event of parturition, or to simulate a delivery which had never taken place; in either of such cases the importance of medical testimony is sufficiently obvious. In cases of alleged infanticide, the practitioner is always required to examine the supposed mother, and to give his opinion as to the fact of her having been recently delivered: and his report has not only very frequently acquitted the prisoner, but in some cases has rescued the innocent, but unfortunate, female from the horror and disgrace of a public trial.

The signs from which the judgment of the midwife is to be deduced may be comprised under the following general and particular heads, to each of which we shall successively direct our attention, and endeavour to establish the degree of validity to which they are singly or jointly entitled.

1. The face is pale, the eye sunken, and surrounded by a purplish or dark brown-coloured ring; the pulse is full and undulating; the skin soft, supple, rather warmer than ordinary, and covered with a moisture which has a peculiar and somewhat acid odour.

2. The breasts are swelled, are harder than usual, and painful; and, in some cases, lumpy to the touch, and emit, by pressure or suction, a lactiform fluid. The nipples are thicker; and the areola, by which they are surrounded, is widened in extent, and darkened in colour.

3. The abdomen is flaccid, and its skin lies in folds, and is traversed in various directions with shining, reddish, and whitish lines, and light-coloured broken streaks, which especially extend from the groins and the pubes towards the navel.

4. There is an extraordinary swelling and tumefaction of the external parts of generation; sometimes the anterior margin of the perineum is lacerated, or it is very lax, from the distention which it has undergone.

5. The vagina is preternaturally distended; the orifice of the uterus is soft and open, and capable of admitting the point of the finger without difficulty, as if a late discharge had been made from it. The womb itself, not having properly collapsed, and resumed its natural shape and dimensions, may be felt through the parietes of the abdomen, voluminous, firm, and globular, contracting and expanding under the hand, on pressure.

6. A discharge of serous fluid mingled with blood from the vagina, called the *lochia*, continues from five to thirty-five days after delivery: it differs from the common menstrual flux in being paler, but more particularly in its peculiar and characteristic sour odour. At first this discharge is decidedly sanious, but in a few days it becomes of a much paler and of a brownish, or dirty-green, hue, so as to acquire the common term of *green waters*.

The relative value which each of the signs possesses, will be better appreciated after we have considered the diseases whose effects may resemble them; but, as a general principle, we are anxious to enforce the necessity of always considering the consecutive signs of parturition collectively, and not individually. Under such circumstances, the practitioner can never be betrayed into an erroneous conclusion; for, as Professor Chaussier has remarked, "no disease or affection, besides parturition, can possibly produce the whole series of signs above described." * * *

In the "Causes Celèbres" there is an account of a girl, who, although a virgin, suckled an infant; and, in the Sloane collection of manuscripts in the British Museum, a case of a woman is related, who, although she had not borne children for more than twenty years, actually suckled her grand-children, one after the other, at the age of sixty-eight! But, what is still more extraordinary, instances have occurred where men have been able to perform this duty. The Bishop of Cork has related a case in which a man suckled his child after the loss of his wife; and in the Personal Narrative of Humboldt we have an analogous instance. * * *

6. *Can we determine by any signs whether a woman has ever borne a child, although at a period remote from that of the examination?*

The following are the principal indications of a woman having been delivered at a distant period; but, in offering them to the attention of the practitioner, it is necessary to observe, that singly they can furnish but very slender evidence; and, should they even all occur, they must be regarded as affording only a strong presumption of the fact.

1. The orifice of the womb has not its conic figure; its lips are unequal; and it is more open than in those who have never borne children.

2. There is a roughness of the abdomen, the parietes of which are also more expanded and pensile.

3. There are small white and shining lines running on the surface of the abdomen.

4. The breasts are more flaccid and pendulous, and the lines on their surface are white and splendid.

5. The nipples are prominent, and the colour of their disks brown.

9. *Is super-fœtation possible, and under what circumstances, and at what period of gestation, can a second conception take place?*

The term *super-fœtation* implies that a second impregnation may take place, whilst a child is in utero.

There are perhaps few questions relating to the subject of conception, that have given origin to more rigorous controversy; and, indeed, its important judicial bearings render it a subject of greater interest than it could ever have become intrinsically as a mere object of abstract speculation. Let us, for the sake of illustration, suppose the following case:—A woman loses her husband suddenly, *tenant in tail male*, a month after marriage, and at a little more than eight months after his decease she is delivered of a perfect female child, and at nine months she declares that she is delivered of another infant, which is a male. The heir at law, who has entered, contests the fact of this latter birth: the question therefore to be determined is, whether such an event is compatible with the known laws of utero-gestation.

The best authenticated case of super-fœtation that has occurred in our own times, is that communicated to the College of Physicians by Dr. Maton:—Mrs. T—, an Italian lady, remarkable for her fecundity, was delivered of a male child at Palermo, on the 12th of November, 1807, under very distressing circumstances, having been dropt on a bundle of straw in an uninhabited room at midnight; and, although the infant at the time of his birth had every appearance of health, he lived only nine days: on February the 2d, 1808, (not quite three calendar months from the preceding accouchement,) Mrs. T— was delivered of another male infant, completely formed, and apparently in perfect health; the child, however, fell a victim to the measles at the age of three months. Dr. Granville, in a paper entitled "On the Malformation of the Uterine System," takes occasion to observe, with respect to the above case, that "it merely goes to prove the occasional co-existence of separate ova in utero, and proves nothing further. The lady, whose prolific disposition is much descanted upon in that paper, and with whom twin-cases were a common occurrence," continues Dr. Granville, "was delivered of a male child some time in November, 1807, '*under circumstances very distressing to the parents, and on a*

*bundle of straw;*' and again, in February 1808, of another male infant, '*completely formed!*'—mark the expression, for it was not made use of in describing the first. The former died, 'without any apparent cause,' when nine days old; the other lived longer. Now, can we consider this otherwise than as a common case of twins, in which one of the fœtuses came into the world at the sixth, and the other at the ninth month of pregnancy, owing to the ova being quite distinct and separate? Had this not been the case, the distressing circumstances, which brought on the premature contraction of the womb, so as to expel part of its contents in November, as in the simplest cases of premature labour, would have caused the expulsion of the whole, or, in other words, of both ova, in that same month; and we should not have heard of the second accouchement in the following February, which led the author of the paper in question to bring the case forward as one of superfœtation, in opposition to what he has called 'the scepticism of modern physiologists.' Had it been proved that the child, of which the body in question was delivered, had reached its full term of utero-gestation in November, and that she had brought forth another child in one, two, or three months afterwards, of equally full growth, then a case something like super-fœtation would have really occurred, and scepticism would have been staggered."—In consequence of the doubts thus expressed by Dr. Granville, the author of the present work, actuated only by a desire after truth, applied to Dr. Maton for a further explanation of those particular points upon which the merits of the case would seem to turn; and he is thus enabled to clear up the doubts which might be supposed to embarrass its history. The fact is, that *both the children were born perfect*, the first therefore could not have been a six-months' child; and, with respect to the *distressing circumstances* which attended the delivery, Dr. Granville appears to have fallen into an important error: he speaks of them as having "brought on the premature contraction of the womb, so as to expel part of its contents in November;" whereas, upon referring to the particular expressions used by Dr. Maton in the paper alluded to, we shall soon perceive that they by no means support the assumption of the labour having been premature, nor that it was brought on by distressing circumstances: on the contrary, we find, upon further inquiry, that the distressing circumstances to which the author alludes were the natural consequence, not the active cause, of the labour. Indeed the fact, as we learn from Dr. Maton, stood thus:—the lady could not obtain better accommodation at the time; that the labour, although quick, was not sudden, for the accoucheur was already in attendance; and that it was not premature, for the natural period of utero-gestation was supposed to have been completed. We must not omit to state that all the particular circumstances of the case were communicated to Dr. Maton by the husband of the lady; and, as he could not have had any particular theory to maintain, or any private interest to serve, there cannot exist any good reason for questioning the veracity of his testimony, or the justness of our conclusions.

Those physiologists who deny the possibility of super-fœtation, among whom we find some of the most celebrated names, assert that one conception can never supervene another in the same woman, because the os uteri is closed by coagulable lymph, and the entrance to the fallopian tubes is obstructed by the decidua uteri soon after conception, and therefore that the semen can never find its way to the internal organs of generation, so as to impregnate a second ovum. This opinion is fortified by the well-known aphorism of Hippocrates, "οκοσαὶ εν γαστρὶ εχουσὶ; τουτεων δε στομαλων υστερων ξυμμεμυκεν." Galen also, quoting Herophilus, says, "Ne specilli quidem mucronem admittere uteros antequam mulier pariat; præterea ne vel minimum quidem hiscere ubi conceperint." Neither Galen, however, nor Ætius, nor Paulus Ægineta, make any mention of superfœtation; a circumstance upon which the opponents of the doctrine lay considerable stress. Avicenna alludes to it, but for the purpose of expressing his disbelief in its possibility. Hebenstreit and Ludwig have also expressed very strong opinions upon the subject: the former of whom observes, "Nullæ fere observationes extra omnem dubitationem positæ superfœtationem confirmant." Baudelocque is equally hostile to such a belief. But it may be said that the argument founded on the entire closure of the uterus is quite gratuitous; many authorities might be cited who disavow the fact. We have already adduced the opinion of Haller upon this point: besides, we are sufficiently acquainted with the manner in which impregnation is effected, to authorise any deductions from our hypothesis? We are completely ignorant in what way the male semen arrives at the internal organs: nay, we are not even convinced that its direct transmission to the ovaria is essential to fecundation; it is possible that these organs may be stimulated by sympathy with the vagina. Parsons opposes another argument to the doctrine of super-fœtation: it is, says he, impossible, because the fallopian tubes become, after conception, too short to embrace the ovaria; but this opinion is successfully combated by Haller. The cases which have been cited to illustrate the phenomenon of super-fœtation, are regarded by those who oppose the doctrine as instances in which a plurality of children has existed, and in which one of the following circumstances have occurred, viz.

1. The fœtus has prematurely died, but has remained in utero with the living child, to the full period of utero-gestation.

2. The descent of the ova into the uterus from the ovarium has not observed the same order of time; one being more slowly evolved than another, although both might have been fecundated by the same coitus.

This latter was the favourite idea of Celoni: "I am therefore decidedly of opinion, (says he,) that this superfœtation is no other than a later development of a fœtus contemporaneously generated."

We have thus presented the reader with a review of the different arguments which have been adopted by the partisans and opponents of this celebrated doctrine; and we have cited copious authorities, with a view to enable the student to pursue the investigation to any extent which may be commensurate with his notions of its importance. We shall now conclude by observing that the following occurrences are essential to constitute a case of super-fœtation.

1. The pregnant woman must bear two children, each of a distinct age.

2. The delivery of these children must take place at different times, with a considerable interval between each.

3. The woman must be pregnant and a nurse at the same time.

11. *Under what circumstances, and by what means, is it morally, legally, and medically proper, to induce premature labour?*

That premature labour may be induced by a mechanical operation, is too well known to the practitioner in midwifery to require any explanation in this place; while, in a work calculated for circulation beyond the confines of the profession, it would be obviously imprudent to enter into any minute details. It becomes our duty, however, to state, that, in those cases of distorted pelvis through which a full-grown fœtus cannot pass without mutilation, the operation may be performed with perfect safety, and with equal advantage both to the child and to the mother. We are informed by Dr. Denman, that there was, in 1756, a consultation of the most eminent men in London at that time, to consider of the moral rectitude of, and advantages which might be expected from, this practice, which met with their general approbation. The morality of this mode of practice, however, says Dr. Merriman, has been doubted by many other persons, but probably for want of considering the question in a proper point of view: for the proposal was, that labour should be prematurely induced *in those cases only* where it had been *decidedly proved* that the pelvis was so much contracted in its dimensions, as to render it impossible for a full-sized fœtus to pass undiminished; and it is supposed that this proceeding, while it affords a chance of preserving the child, does not much implicate the life of the mother. Mr. J. Barlow has given us the result of an extensive practice in inducing premature labour in cases of distorted pelvis, from which it appears that he has had recourse to this method of delivery *eighteen* times, in five women, all of whom had been previously delivered once, or oftener, by the crotchet, and that premature labour occurred spontaneously once in two of this number. All the women recovered; a circumstance which adds a further confirmation to the opinion that the life of the parent is exposed to very little hazard in this way. Of the children thus brought into the world, six were dead and twelve were born alive; of which some died soon after birth, one lived ten months, and five were living at the time the account was published. Mr. Barlow's method consists in exciting premature labour early in the seventh month of pregnancy. Dr. Hull, well known for his controversial zeal on these subjects, has offered some remarks so judicious and important, that it would be an act of injustice to withhold them from the reader. "The propriety of inducing premature labour, (says he,) in any deformed woman, can rarely, if ever, be determined upon before the crotchet has been found indispensably necessary, and actually employed in a previous labour: indeed, unless the contraction of the tube or canal of the pelvis be very considerable, and pretty accurately ascertained, it will scarcely be justifiable in any case to have recourse to this practice in all the subsequent pregnancies, until the woman has been delivered a second or third time by the crotchet; for it has happened in a very great number of instances, that a woman who has been delivered of her first child by the perforator and crotchet, has been afterwards delivered of one or more living children at the full time. This observation is made not to discountenance the inducing of premature labour, but to prevent the abuse of it." Dr. Merriman, whose extensive practice and generally acknowledged judgment stamp a peculiar value upon his opinions, has also pointed out the limitations and cautions which he deems necessary to be observed, to render this operation safe and eligible; and he concludes by observing, that "a regard to his own character should determine the accoucheur not to perform this operation, unless some other respectable practitioner has seen the patient, and has acknowledged that the operation is advisable."

12. *What circumstances will justify the Cæsarean operation, and of what value is the section of the symphysis pubis, or sigaultian operation?*

Instrumental delivery resolves itself into three classes—

1. Where neither the mother nor the child is of necessity injured, as by the use of the *forceps* and *lever*.

2. Where the mutilation of the child is the principal object, as by the *perforator* and *crotchet*.

3. Where the mother is wounded, as in the *Cæsarean* and *Sigaultian operations*.

It is of the latter class we have now to speak.

In this country the operation has been generally fatal. A very extraordinary case is, however, stated to have occurred in Ireland, and, however incredible the story may appear, says Dr. Merriman, there seems no reason to doubt its truth: it is related by Mr. Duncan Stewart, surgeon, in Dungannon, who saw the patient some days after the operation; and the account is confirmed by Dr. Gabriel King, of Armagh, who says that he drew out the needles which the midwife had left to keep the lips of the wound together. The patient's name was Alice O'Neil, and the operator was an illiterate midwife, one Mary Dunally; the instrument used was a razor, with which she first cut through the containing parts of the abdomen, and then the uterus. "She held the lips of the wound together with her hand, till some one went a mile and returned with silk and the common needles which tailors use; with these she joined the lips, in the manner of the stitch employed ordinarily for the hare-lip, and dressed the wound with whites of eggs." The woman recovered in twenty-seven days.

It has often been an object of inquiry, why this operation should have been more successful upon the continent than in this country? The answer to the question is obvious and satisfactory. In this country we have only had recourse to it as an operation of necessity, where we can neither accomplish the delivery by diminishing the bulk of the child, nor by any of the other resources already explained; whereas the practitioners of France, and the other states on the continent of Europe, perform it not only as an operation of necessity, but as one of election, in cases where the mother may confessedly be delivered with safety, by sacrificing the life of the fœtus: it would also appear that, in general, they have recourse to the operation before the patient has suffered very much from the continuance of labour. How greatly this circumstance is capable of influencing the success of a surgical operation, we have a satisfactory demonstration in the history of that for hernia, and in which Mr. Bell informs us the French were formerly more fortunate, because they proceeded more early to the operation than the surgeons of almost any other nation. It deserves notice that the religious tenets of different countries appear to have influenced the popularity of the Cæsarean section: it is easy to suppose that, in those Catholic nations where a belief exists of the necessity of baptism to secure the eternal happiness of the infant, the mother would become a willing sacrifice to make her offspring a Christian.

In delivering our opinion upon the propriety of performing the Cæsarean section in this kingdom, we should say that there are cases in which it is the bounden duty of the accoucheur to proceed without delay; and such appears to have been that described by Dr. Merriman, of which the pelvis in the museum of Mr. Charles Bell is a sufficient proof; for so extreme is the distortion, that a marble, measuring less than one inch in diameter, cannot be made to pass through it in any direction. In this case, and some others of a similar nature, the Cæsarean section was the only means of preserving the child. We are of opinion, however, that the operation ought never to be performed where by embryulcia the child can be extricated; and, although circumstances of inheritance should induce the husband to entertain a feeling like that which animated Henry VIII, the practitioner has but one broad line of duty to observe,—to save, if possible, the mother and child; but, where this is impossible, to feel no hesitation in sacrificing the life of the latter. In the event of a woman, near the full time of pregnancy, dying undelivered, the Cæsarean operation ought always to be performed with as little loss of time as possible; since by this measure a chance of preserving the child will be afforded: and Dr. Merriman states that several cases of such an operation, after the death of the mother, have been recorded, with the desired effect of saving the infant.

